# Evaluation of the Safety, Tolerability, and Pharmacokinetic Profiles of TP0473292 (TS-161), A Prodrug of a Novel Orthosteric mGlu2/3 Receptor Antagonist TP0178894, in Healthy Subjects and Its Antidepressant-Like Effects in Rodents

**DOI:** 10.1093/ijnp/pyab062

**Published:** 2021-09-17

**Authors:** Mai Watanabe, Brian Marcy, Ayano Hiroki, Hirotaka Watase, Kohnosuke Kinoshita, Michihiko Iijima, Toshiyuki Marumo, Carlos A Zarate, Shigeyuki Chaki

**Affiliations:** 1 Taisho Pharmaceutical R&D Inc., Morristown, New Jersey, USA; 2 Taisho Pharmaceutical Co., Ltd., Tokyo, Japan; 3 National Institute of Mental Health, National Institute of Health, Bethesda, Maryland, USA

**Keywords:** Cerebrospinal fluid, mGlu2/3 receptor antagonist, TP0178894, TP0473292, treatment-resistant depression

## Abstract

**Background:**

TP0473292 (the active ingredient of TS-161) is a prodrug of a novel metabotropic glutamate (mGlu) 2/3 receptor antagonist being developed for the treatment of patients with depression. This study evaluated the safety, tolerability, and pharmacokinetics of orally administered TS-161 in healthy subjects.

**Methods:**

This was a first-in-human, phase 1, randomized, double-blind, placebo-controlled, single-ascending dose (15–400 mg TS-161) and 10-day multiple-ascending dose (50–150 mg TS-161) study in healthy subjects, conducted from June 2019 through February 2020. Plasma and urine concentrations of the prodrug and its metabolites, and cerebrospinal fluid (CSF) concentrations of the active metabolite TP0178894 were measured to evaluate the pharmacokinetic profiles after oral administration of TS-161.

**Results:**

Following single and multiple doses, TP0473292 was extensively converted into its active metabolite TP0178894. Plasma concentrations of TP0178894 reached peak (C_max_) within 5 hours post dose and declined with a t_1/2_ <13 hours. Plasma exposures of TP0178894 increased with increasing dose. TP0178894 penetrated into CSF and reached a C_max_ of 9.892 ng/mL at a single dose of 100 mg, which was comparable with IC_50_ values of antagonist activity at mGlu2/3 receptors. The most frequently observed adverse events that showed exposure-related incidence during the study were nausea, vomiting, and dizziness.

**Conclusions:**

The mGlu2/3 receptor antagonist prodrug TP0473292 is safe and well-tolerated, is orally bioavailable in humans with extensive conversion into the active metabolite TP0178894 with sufficient CSF penetration to exert the anticipated pharmacological effects, and is a promising candidate for further clinical development in treatment of patients with depression.

**Significance Statement**

The blockade of mGlu2/3 receptors has emerged as an attractive approach to treat depression based on several preclinical studies that showed antidepressant-like effects similar to those seen in (*R,S*)-ketamine treatment in animal models, which demonstrated a rapid-acting antidepressant effect in treatment-resistant depression (TRD) patients. This research report presents the in vitro and antidepressant-like profiles of TP0473292 (TS-161), a novel orthosteric mGlu2/3 receptor antagonist prodrug, and its active metabolite TP0178894 in animal models. Furthermore, this first-in-human, single and multiple ascending dose study of TS-161 demonstrated the safety profile as well as a successful prodrug strategy with rapid conversion of TP0473292 into TP0178894, which penetrated into the cerebrospinal fluid with the concentrations predicted to be sufficient to act on presumed drug targets to exert the anticipated antidepressant effects, when administered orally in healthy subjects. These results warrant further clinical development of TS-161 in patients with depression, including TRD.

## Introduction

Abnormalities of glutamatergic transmission have been implicated in several psychiatric disorders, and agents acting on glutamatergic systems have gained much attention for drug discovery of treatments for psychiatric disorders (Sanacora et al., 2008; Hashimoto, 2014). Glutamate exerts a variety of physiological functions via 2 major classes of receptors, ionotropic glutamate receptors and metabotropic glutamate (mGlu) receptors (Nakanishi, 1992), which consist of 8 subtypes (mGlu1–mGlu8) that are classified into 3 major groups (group I–III). Among mGlu receptors, growing evidence suggests that group II mGlu receptors (mGlu2 and mGlu3 receptors), which have major roles in the regulation of glutamatergic transmission within the cortical and limbic systems (Wright et al., 2013), may serve as potential therapeutic targets for neuropsychiatric disorders, including schizophrenia (Chaki, 2010; Stansley and Conn, 2018), depression (Chaki, 2017), anxiety (Swanson et al., 2005), and cognitive impairment (Kim et al., 2014).

Particularly, blockade of the mGlu2/3 receptors has emerged as an attractive approach to treat depression based on several preclinical studies (Chaki, 2017; Witkin, 2020). This idea stems mainly from animal study results showing that mGlu2/3 receptor antagonists exert antidepressant-like effects similar to those seen in (*R,S*)-ketamine treatment (Chaki, 2004, 2017; Joffe and Conn, 2019; Witkin, 2020; Pałucha-Poniewiera et al., 2021), which demonstrated a rapid-acting antidepressant effect in treatment-resistant depression (TRD) patients (Zarate et al., 2006). Notably, a nasal spray (Spravato) that delivers esketamine, a stereoisomer of (*R,S*)-ketamine, was approved by the US Food and Drug Administration as an adjunct therapy in conjunction with an oral antidepressant for the treatment of TRD patients in 2019 and for depressive symptoms in adults with major depressive disorder (MDD) with acute suicidal ideation or behavior in 2020. In addition to the antidepressant profiles, it was previously reported that both mGlu2/3 receptor antagonists and (*R,S*)-ketamine share mechanisms underlying the antidepressant-like effects on synaptic levels (Koike et al., 2011, 2013a; Pałucha-Poniewiera et al., 2019) and network levels (Fukumoto, 2016; Chaki and Fukumoto, 2019; Zanos et al., 2019). Importantly, although (*R*,*S*)-ketamine produces ancillary effects, including abuse liability and psychotomimetic effects (Iadarola et al., 2015), rodent studies suggest that treatment with mGlu2/3 receptor antagonists would be more tolerable and without the (*R*,*S*)-ketamine treatment associated side effects (Witkin, 2020). These findings indicate that mGlu2/3 receptor antagonists can be a useful pharmacotherapy for treatment of MDD patients, including those with TRD.

**Figure 1.**
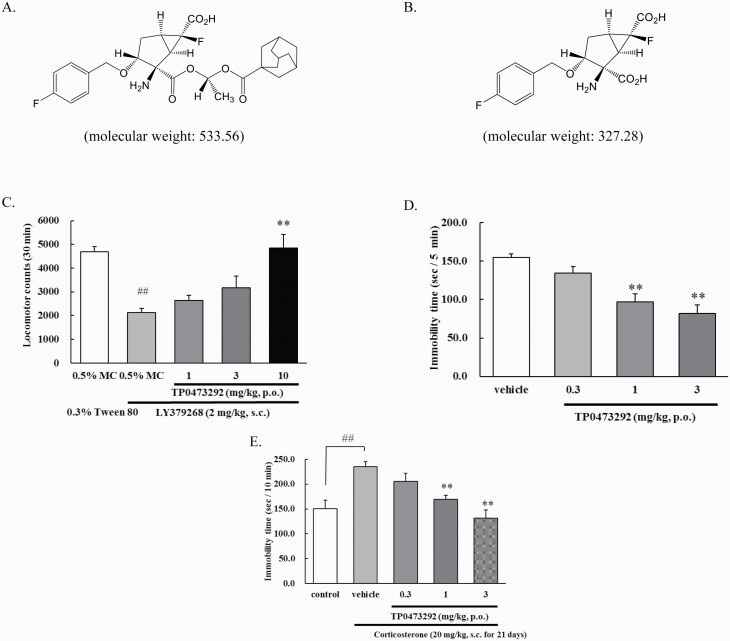
Chemical structures of TP0473292 and TP0178894, and in vivo pharmacological profiles of TP0473292. (A) Chemical structure of TP0473292; (B) Chemical structure of TP0178894; (C) Effect of oral administration (p.o.) of TP0473292 on mGlu2/3 receptor agonist, LY379268-induced hypolocomotion in rats. Locomotor activity was measured for 30 min after subcutaneous (s.c.) administration of LY379268. Data represent mean ± SE (n = 8/group). ##*P* < .01 compared with 0.5% methyl cellulose 400 (MC) group (Student’s t-test), **P < .01 compared with LY379268-treated 0.5% MC group (Steel’s test); (D) Effect of oral administration (p.o.) of TP0473292 on immobility time in the forced swimming test of rats. Immobility time during the forced swimming test was measured for 5 min. Data represent mean ± SE (n = 9 – 10/group). **P < .01 compared with vehicle group (Dunnett’s test); (E) Effect of oral administration (p.o.) of TP0473292 on depressive-like behavior of corticosterone (CORT) treatment model of rats. Depressive-like behavior (increased immobility) was induced by treating rats with CORT (20 mg/kg) subcutaneously (s.c.) for 21 days, and the forced swimming test was conducted 2 days after the final CORT injection. Immobility time during the forced swimming test was measured for 10 minutes. Data represent mean ± SE (n = 8/group). ##*P* < .01 compared with control group (Student’s *t* test), **P < .01 compared with vehicle-treated group (Dunnett’s test).

Nonetheless, an mGlu2/3 receptor negative allosteric modulator, decoglurant, failed to show efficacy in patients with partially refractory MDD (Umbricht et al., 2020). Several considered reasons for this failure include lack of data to validate target engagement in humans (Umbricht et al., 2020) and a failure to show (*R*,*S*)-ketamine–like profiles in rodents (Sheffler et al., 2019). Therefore, other human proof-of-concept studies using mGlu2/3 receptor antagonists with appropriate pharmacokinetic (PK) and pharmacological profiles are needed to clarify the potential of mGlu2/3 receptor antagonists for the treatment of depression. We synthesized (1*R*,2*R*,3*R*,5*R*,6*R*)-2-({(1*S*)-1-[(adamantane-1-carbonyl)oxy]ethoxy}carbonyl)-2-amino-6-fluoro-3-[(4-fluorophenyl)methoxy]bicyclo[3.1.0]hexane-6-carboxylic acid (TP0473292), a prodrug of an orthosteric mGlu2/3 receptor antagonist, (1*R*,2*R*,3*R*,5*R*,6*R*)-2-amino-6-fluoro-3-[(4-fluorophenyl)methoxy]bicyclo[3.1.0]hexane-2,6-dicarboxylic acid (TP0178894) (Nakazato et al., 2004) (Figure 1A–B). Here, we report in vitro and antidepressant profiles of TP0473292 and its active metabolite, TP0178894, as well as the safety, tolerability, and PK of single- and multiple-ascending doses of TS-161, the investigational product containing TP0473292 as the active ingredient, in healthy subjects.

## METHODS

### Preclinical Pharmacology Studies of TP0178894 and TP0473292

#### Materials

**—**Male Sprague-Dawley rats (7 weeks; Charles River, Yokohama, Kanagawa, Japan) were used for behavioral tests except for a corticosterone (CORT) treatment model in which male Sprague-Dawley rats (4 weeks old at the beginning of the experiments; Charles River, Yokohama, Japan) were used. The animals were maintained under controlled temperature (23°C ± 3°C) and humidity (50% ± 20%) conditions under a 12-hour-light/-dark cycle (lights on at 7:00 am). Food and water were provided ad libitum. All the studies were performed according to the guidelines of the Taisho Pharmaceutical Co., Ltd. Animal Care Committee and met the Japanese Experimental Animal Research Association standards, as defined in the Guidelines for Animal Experiments. TP0473292, TP0178894, and 1-*O*-(tricyclo[3.3.1.1^3,7^]decane-1-carbonyl)-*β*-D-glucopyranuronic acid (TP0478768) were synthesized at the Research Center of Taisho Pharmaceutical Co., Ltd. Adamantane-1-carboxylic acid (TP0037870) and LY379268 were purchased from Tokyo Chemical Industry Co., Ltd. (Tokyo, Japan) and Abcam plc. (Cambridge, UK), respectively. For in vivo studies, TP0473292 was suspended in 0.5% methyl cellulose 400, and LY379268 was dissolved with 0.3% Tween 80.

### Methods for in Vitro and in Vivo Pharmacological Studies

#### Antagonist Activity for mGlu2 and mGlu3 Receptor

**—**Antagonist activity of TP0178894 for mGlu2 and mGlu3 receptors was evaluated by [^35^S]GTPγS binding to recombinant human or rat receptors. Briefly, membrane preparations (10 μg protein/assay) in 20 mmol/L HEPES buffer (pH 7.4) containing 100 mmol/L NaCl, 10 mmol/L MgCl_2_, 10 μg/mL saponin, and 0.1% bovine serum albumin were incubated with guanosine diphosphate (8.4 μmol/L), glutamate (20 μmol/L for mGlu2, 1 μmol/L for mGlu3), [^35^S]GTPγS (0.15 nmol/L), and various concentrations of TP0178894 at 30°C for 60 minutes. The reaction was terminated by rapid filtration through a UniFilter GF/C microplate after which the filters were washed with ice-cold 20 mmol/L HEPES buffer (pH 7.4). Non-specific binding was determined in the absence of glutamate.

#### Affinity for Human mGlu2 and Human mGlu3 Receptor

**—**Affinity of TP0178894 and TP0473292 for human mGlu2 and human mGlu3 receptors was evaluated by [^3^H]LY341495 binding to recombinant human or rat receptors. Briefly, membrane preparations (1 μg protein/assay for human mGlu2, 2 μg protein/assay for human mGlu3) in 10 mmol/L potassium phosphate buffer (pH 7.6) containing 100 mmol/L potassium bromide and 0.1% bovine serum albumin were incubated with [^3^H]LY341495 (1 nmol/L) and various concentrations of TP0178894 or TP0473292 on ice for 60 minutes. The reaction was terminated by rapid filtration through a UniFilter GF/C microplate presoaked with 0.3% polyethyleneimine, after which the filters were washed with ice-cold 10 mmol/L potassium phosphate buffer (pH 7.6) containing 100 mmol/L potassium bromide. Non-specific binding was determined in the presence of 1 mmol/L glutamate.

#### Locomotor Activity

**—**Rats were placed individually in transparent, open-topped acrylic cages (47 × 28 × 30 cm), and spontaneous locomotor activity was recorded using a SCANET apparatus (Melquest Ltd, Toyama, Japan) placed in a sound-proof box. To investigate the effects of TP0473292 on spontaneous locomotor activity, TP0473292 (1, 3, 10 mg/kg) was administered orally 60 minutes before the start of measurement, and locomotor activity was recorded for 60 minutes. To investigate the effects of TP0473292 on LY379268-induced hypolocomotion, TP0473292 (1, 3, and 10 mg/kg) was administered orally 2 hours prior to the start of the measurement, LY379268 (2 mg/kg) was administered subcutaneously 20 minutes before the start of the measurement, and locomotor activity was recorded for 30 minutes.

#### Forced Swimming Test

**—**A forced swimming test was conducted according to a previously reported method (Chaki et al., 2004). The acrylic cylinders (diameter 18 cm, height 50 cm) containing water (25°C ± 1°C, 30 cm deep) were used. TP0473292 (0.3, 1, 3 mg/kg) was administered orally 15 minutes after pretest (15 minutes pre-swim session) and 60 minutes prior to the test (5-minute test session approximately 24 hours after pre-swim). During the test session, time engaged in immobility, which was defined as the behavior of floating in water without struggling or moving but to keep the head above the water, was measured. After the swim test, the plasma, cerebrospinal fluid (CSF), and brain samples were quickly collected for follow-up PK studies.

#### Repeated CORT Treatment Model

**—**The effect of TP0473292 in the CORT treatment model was assessed according to a previously reported method (Koike et al., 2013b). Rats were s.c. administered daily with CORT (20 mg/kg), and depressive-like behaviors induced by CORT treatment was determined by using the forced swimming test. The forced swimming test was conducted 2 days after the final CORT injection, as described above, except a pre-swim session was not conducted and the duration of the test session was 10 minutes. TP0473292 (0.3, 1, 3 mg/kg) was administered orally twice at 24 hours pre-swim and again at 60 minutes prior to the test session.

#### PK Analyses

**—**Immediately after the swimming test, the rats were killed, and blood, CSF, and brain (cerebrum) samples were collected. Each sample was extracted using protein precipitation and was subsequently analyzed to determine the TP0178894 levels using a validated liquid chromatography/tandem mass spectrometry method on a QTRAP6500 instrument (Sciex, Framingham, MA, USA).

#### Statistical Analyses

**—**For in vitro studies, IC_50_ values were determined from each concentration-response curve by non-linear least-squares curve-fitting procedure, using the SAS 9.2. IC_50_ values of affinities for mGlu2 and mGlu3 receptors were converted to K_i_ values using the Cheng-Prusoff equation. For the in vivo studies, data were analyzed by applying the F-test or Bartlett’s test to examine the homogeneity in variance, followed by Student’s *t* test (for comparisons between 2 groups) and Dunnett’s test or Steel’s test (for multiple comparisons). All the statistical analyses were performed using SAS system version 8.2 (SAS Institute Japan, Tokyo, Japan). A value of *P* < .05 was considered significant.

The materials and methods for the in vitro pharmacology studies evaluating antagonist activities for other mGlu receptors and selectivity over other receptors, transporters, and ion channels are described in the supplementary Material.

### Clinical Study Design

The phase 1 first-in-human study was composed of 3 parts: Part A (SAD: single-ascending dose), Part B (CSF sampling), and Part C (MAD: multiple-ascending dose) (Table 1). The study employed a randomized, double-blind, placebo-controlled, parallel group design (Parts A and C) to evaluate the safety, tolerability, and PK of TS-161 as oral capsules in healthy subjects. Part B, which was primarily designed to evaluate CSF PK, was open-label and no subjects received placebo. The study was conducted at a single US site from June 2019 to February 2020 (ClinicalTrials.gov identifier: NCT03919409). The study was approved by IntegReview IRB (Austin, TX) and conducted in accordance with the Declaration of Helsinki and the International Council on Harmonisation Guideline for Good Clinical Practice. Subjects provided written consent before participating in the study.

**Table 1.** Clinical Study Design

Part		A (SAD)					B (CSF)	C (MAD)		
Cohort		1	2	3	4	5	6	7	8	9
Dose (mg)		15	50	100	200	400	100	50	150	100
Meal condition		Fasted	Fasted/Fed	Fasted	Fasted	Fasted	Fasted	Fed	Fed	Fed
N	TS-161	6	6	6	6	6	6	6	6	6
	Placebo	2	2	2	2	2	0	2	2	2

Abbreviations: CSF, cerebrospinal fluid; MAD, multiple-ascending dose; SAD, single-ascending dose.

Part A subjects were randomized in a 3:1 ratio to each of 5 cohorts to receive a single dose of either TS-161 (n = 6) in doses ranging from 15 mg (Cohort 1) to 400 mg (Cohort 5), or placebo (n = 2). All cohorts were dosed under fasted conditions, except Cohort 2, which was first dosed in a fasted condition, then in a fed condition to evaluate food effects. Part B subjects received a single dose of 100 mg TS-161 under fasted conditions (Cohort 6). Part C subjects were randomized in a 3:1 ratio to each of 3 cohorts to receive once-daily doses of TS-161 of either 50 mg (Cohort 7), 150 mg (Cohort 8), 100 mg (Cohort 9) (n = 6), or placebo (n = 2) under fed conditions for 10 days. The site’s unblinded statistician generated a randomization schedule. Subjects were observed as inpatients from Day −1 until 48 hours following the last drug administration.

### Subject Selection

Healthy males and female subjects who were not pregnant or breastfeeding, aged 18 to 55 years inclusive, body weight ≥45 kg, with a body mass index of 18–30 kg/m^2^ were eligible for the study. Exclusion criteria included, but were not limited to clinically significant abnormal cardiac examinations, hematology, biochemistry, or urinalysis; significant history or presence of hepatic, renal, cardiovascular, or pulmonary disease; history or presence of psychiatric or neurological disease or condition; or use of *N*-methyl-D-aspartate receptor modulators prior to the study.

### Safety Assessments

Safety and tolerability were assessed from adverse events (AEs), vital signs, 12-lead electrocardiograms, clinical laboratory tests (hematology, biochemistry, and urinalysis), the Brief Psychiatric Rating Scale (BPRS) (Overall and Gorham, 1988), the Coding subtest of The Repeatable Battery for the Assessment of Neuropsychological Status (RBANS) (Randolph et al., 1998), the Clinician Administered Dissociative States Scale (CADSS) (Bremner et al., 1998), physical and neurological examinations, and the Columbia-Suicide Severity Rating Scale (Posner et al., 2011), all of which were conducted at scheduled timepoints at pre and post dose.

### PK Assessments and Bioanalytical Methods

Blood, CSF, and urine samples were collected for PK assessment.

Blood samples for the determination of TP0473292, TP0178894, TP0037870 (a metabolite released from the ester side-chain moiety of TP0473292), and TP0478768 (a glucuronide of TP0037870) plasma concentrations were collected in Part A at pre dose, 0.25, 0.5, 0.75, 1, 1.5, 2, 3, 4, 5, 6, 8, 10, 12, 24, 36, and 48 hours post dose. Part C blood samples were collected repeatedly on the first and last days of a 10-day dosing period at the same time points as Part A and at pre dose on Days 2–9. Part B CSF and blood samples were collected at pre dose, 0.5, 1, 1.5, 2, 3, 4, 5, 6, 8, 12, and 24 hours post dose.

Urine samples were collected at 24-hour intervals until 48 hours after the last administration.

Plasma, urine, and CSF samples were analyzed at Toray Research Center, Inc. (Kamakura, Kanagawa, Japan). The samples were quantified for TP0473292, TP0178894, TP0037870, and TP0478768 in plasma and urine and for TP0178894 in CSF using validated liquid chromatography/tandem mass spectrometry methods on a QTRAP5500 or QTRAP6500 instrument (Sciex). In the plasma samples, the lower limits of quantification (LLOQ) for TP0473292, TP0178894, TP0037870, and TP0478768 were 0.1, 0.3, 0.3, and 0.3 ng/mL, respectively, and the respective upper limits of quantification (ULOQ) were 100, 300, 300, and 300 ng/mL. In the urine samples, the LLOQ were 1 ng/mL for TP0473292 and TP0178894, and 3 ng/mL for TP0037870 and TP0478768. The ULOQ were 1000 ng/mL for TP0473292 and TP0178894, and 3000 ng/mL for TP0037870 and TP0478768. In the CSF samples, the LLOQ and ULOQ for TP0178894 were 0.1 ng/mL and 100 ng/mL, respectively. All the analytical runs met the acceptance criteria: At least 2/3 of the total quality control samples and at least 50% at each concentration level were within ±15% of their nominal concentrations for each analytical run.

### Statistics

Placebo-treated subjects from different cohorts were pooled as control groups by condition (fasted or fed) for Part A and as 1 control group for Part C in the summary statistics. Plasma PK parameters were calculated by non-compartmental analysis methods using Phoenix WinNonlin Version 8.0 (Certara USA, Inc., Princeton, NJ). Statistical analysis for BPRS, CADSS, and Coding subtest of RBANS are described in the supplementary Material.

The total number of enrolled subjects was based on practical considerations for a Phase 1 study and not on statistical power.

## RESULTS

### Preclinical Pharmacology Profiles of TP0178894 and TP0473292

The active metabolite TP0178894 showed high affinity (K_i_ values; 4.27 ± 0.22 nmol/L for mGlu2, 2.83 ± 0.09 nmol/L for mGlu3) and potent antagonist activity (IC_50_ values; 23.3 [16.3 – 33.5] nmol/L for mGlu2, 20.9 [12.2 – 35.8] nmol/L for mGlu3) at both human mGlu2 and mGlu3 receptors (Table 2). No species differences in antagonist activity were observed between rat and human (Table 2). In contrast, TP0178894 showed weak or negligible activities at other mGlu receptor subtypes (supplementary Table 1). Moreover, TP0178894 did not have any affinity for 66 different targets (receptors, transporters, and ion channels) at 10 000 nmol/L (supplementary Table 2). Therefore, TP0178894 is a potent and selective antagonist of mGlu2/3 receptors. In contrast, TP0473292, a prodrug of TP0178894, did not have meaningful affinities for either human mGlu2 (K_i_ value; 5,060 ± 394 nmol/L) or mGlu3 receptors (K_i_ value; 5,010 ± 788 nmol/L), nor for 66 different targets (supplementary Table 2) except the NK_1_ and V_1a_ receptors, in that TP0473292 showed more than 50% inhibition at 10 000 nmol/L. Oral administration of TP0473292 showed significant attenuation of mGlu2/3 receptor agonist (LY379268, 2 mg/kg, s.c.) induced hypolocomotion in rats (Figure 1C), indicating in vivo antagonism against mGlu2/3 receptors. In addition, oral administration of TP0473292 significantly reduced immobility time in the rat forced swimming test (Figure 1D), indicating antidepressant-like effects. Dose-dependent concentrations of its active metabolite TP0178894 in the CSF was confirmed in this test (Table 3). It was confirmed that oral administration of TP0473292 (1, 3, and 10 mg/kg) did not affect spontaneous locomotor activity (data not shown). Notably, oral administration of TP0473292 significantly attenuated depressive-like behaviors in a CORT-treated rat model (Figure 1E), proposed to be a model of treatment refractory depression (Iijima et al., 2010; Koike et al., 2013b). Therefore, TP0473292 has antidepressant profiles similar to those of (*R*,*S*)-ketamine in rodents.

**Table 2:** In Vitro Profiles of TP0178894 for mGlu2 and mGlu3 Receptors

	Human mGlu2	Human mGlu3	Rat mGlu2	Rat mGlu3
	IC_50_ or Ki (nmol/L)^*a*^			
Affinity	4.27 ± 0.22	2.83 ± 0.09	N.D.	N.D.
Antagonist activity	23.3 [16.3 to 33.5]	20.9 [12.2 to 35.8]	23.2 [14.8 to 36.3]	27.8 [18.3 to 42.4]

Abbreviations: IC_50_, half maximal (50%) inhibitory concentration; K_i_, inhibition constant; mGlu, metabotropic glutamate; N.D., not determined.

^*a*^Values of affinity were shown as K_i_ values and values of antagonist activity were shown as IC_50_ values. Data (K_i_) represent mean ± SE obtained from 3 independent experiments. Data (IC_50_) represent mean [95% confidence interval] obtained from 3 independent experiments.

**Table 3.** Concentrations of TP0178894 in Plasma, CSF, and Cerebrum After Oral Administration of TP0473292 to Rats

	Concentration of TP0178894		
TP0473292 Dose (mg/kg)	Plasma (ng/mL)	CSF (ng/mL)	Cerebrum (ng/g)
0.3	82.6 ± 21.3	0.706 ± 0.205	1.92 ± 0.475
1	225 ± 92.4	1.87 ± 1.09	5.08 ± 1.30
3	636 ± 158	5.74 ± 2.13	15.0 ± 2.49

Abbreviation: CSF, cerebrospinal fluid.

TP0473292 was administered orally 24 hours and 60 minutes prior to the forced swimming test. After a 5-minute swim session, rats were killed and plasma, CSF, and cerebrum samples were collected for pharmacokinetic study. Data represent mean ± SD (n = 9–10/group).

### Subject Disposition and Demographics

Seventy subjects were enrolled into the clinical study. Fifty-four subjects received TS-161 and 16 subjects received placebo, and 67 subjects completed the study (supplementary Table 3). Three Part C (Cohort 8) subjects prematurely discontinued due to subjects’ request to withdraw related to AEs: 2 subjects in the 150 mg TS-161 group and 1 placebo group subject. Demographic characteristics were comparable among the treatment groups (supplementary Table 4).

### Pharmacokinetics

**Figure 2.**
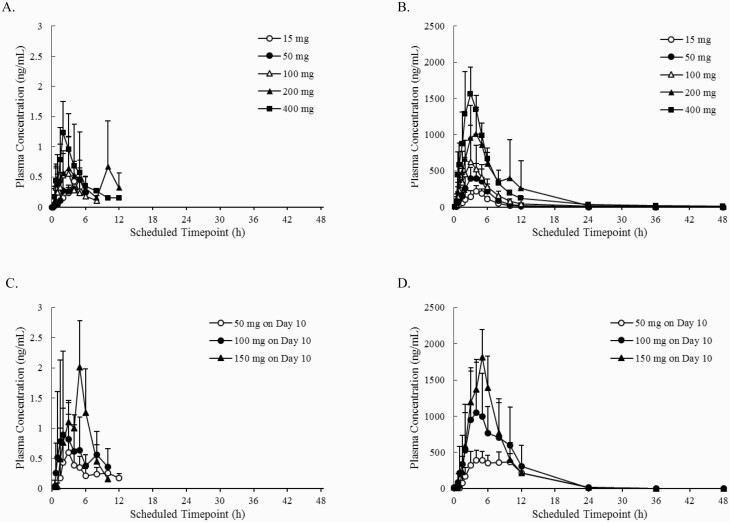
Mean plasma concentration-time profiles of (A) TP0473292 in Part A following single-ascending dose (SAD) under fasted conditions, (B) TP0178894 in Part A (SAD, fasted), (C) TP0473292 on Day 10 in Part C following multiple-ascending dose (MAD) under fed conditions, and (D) TP0178894 on Day 10 in Part C (MAD, fed). Figures are presented as the mean + SD. Figures are presented as the mean + SD (n = 6).

Plasma concentration-time profiles and PK parameters of TP0473292 and TP0178894 following single and multiple doses of TS-161 are shown in Figure 2 and Table 4, respectively. Plasma TP0178894 concentrations reached C_max_ within 5 hours (median), with mean t_1/2_ of less than 13 hours after single and multiple doses, while plasma exposure (C_max_ and area under concentration-time curves [AUCs]) of TP0473292 was minimal at all doses, accounting for approximately 0.1% of C_max_ and AUCs of TP0178894 in a molar concentration. Plasma C_max_ and AUCs of TP0178894 rose with increasing dose in single and multiple doses. Food increased the AUCs of TP0178894 by approximately 70%, resulting in higher TP0178894 exposure at a given dose level in a multiple-dose study conducted under fed conditions compared with single-dose under fasted conditions. Plasma trough concentrations of TP0178894 appeared to reach steady state by 2 days after initiating multiple doses without accumulation during 10 days of daily dosing.

**Table 4.** Plasma Pharmacokinetic Parameters of TP0473292, and Plasma and CSF Pharmacokinetic Parameters of TP0178894 by Treatment

TP0473292 (Plasma)											
Parameter (unit)	Part A (SAD)						Part C (MAD) (Day 10)				
	15 mg Fasted (n = 6)	50 mg Fasted (n = 6)	50 mg Fed (n = 6)	100 mg Fasted (n = 6)	200 mg Fasted (n = 6)	400 mg Fasted (n = 6)	50 mg Fed (n = 6)		100 mg Fed (n = 6)	150 mg Fed (n = 6)	
C_max_ (ng/mL)^*a*^	0.4388 (67.8)	0.5068 (44.0)	0.5123 (52.0)	0.7888 (64.4)	1.132 (46.4)	1.469 (35.9)	0.7438 (77.6)		1.474 (76.7)	2.133^*e*^ (28.4)	
AUC (h·ng/mL)^*a*^,,^*b*^	NC	NC	NC	NC	NC	NC	4.001^*h*^ (NC)		5.337^*g*^ (46.8)	8.073^*f*^ (15.2)	
t_max_ (h)^*c*^	3.00	3.56	5.48	2.50	4.01	2.02	3.03		4.01	5.05^*e*^	
t_1/2_ (h)^*a*^	NC	NC	NC	NC	NC	NC	2.006^*h*^ (NC)		1.350^*g*^ (32.8)	1.178^*g*^ (26.0)	
TP0178894 (plasma and CSF)											
Parameter (unit)	Part A (SAD)						Part B (CSF)		Part C (MAD) (Day 10)		
	Plasma						Plasma	CSF	Plasma		
	15 mg Fasted (n = 6)	50 mg Fasted (n = 6)	50 mg Fed (n = 6)	100 mg Fasted (n = 6)	200 mg Fasted (n = 6)	400 mg Fasted (n = 6)	100 mg Fasted (n = 6)		50 mg Fed (n = 6)	100 mg Fed (n = 6)	150 mg Fed (n = 6)
C_max_ (ng/mL)^*a*^	240.5 (24.2)	484.3 (33.9)	565.2 (20.4)	691.3 (65.9)	1293 (20.4)	1622 (19.6)	820.7 (47.1)	9.892 (27.0)	487.7 (21.0)	1623 (14.8)	1895^*e*^ (13.0)
AUC (h×ng/mL)^*a*^^,^^*b*^	1105^*d*^ (4.2)	2151^*f*^ (45.7)	3568^*d*^ (12.0)	3598 (38.0)	7345^*e*^ (23.2)	9344^*d*^ (20.7)	4890^*e*^ (41.8)	106.5^*f*^ (38.6)	4344 (12.4)	8695 (15.8)	10260^*e*^ (19.3)
t_max_ (h)^*c*^	4.14	4.07	5.00	3.00	4.01	2.99	3.01	7.04	5.00	4.51	4.50^*e*^
t_1/2_ (h)^*a*^	3.039^*d*^ (22.9)	5.693^*f*^ (42.7)	2.976^*d*^ (9.9)	9.339 (39.5)	12.89^*e*^ (20.0)	10.55^*d*^ (33.4)	3.430^*e*^ (19.6)	6.362^*f*^ (19.1)	4.029 (4.6)	7.228^*d*^ (58.7)	5.231^*e*^ (33.8)

Abbreviations: AUC_0-inf_, area under the concentration-time curve extrapolated to infinity; AUC_0-tau_, area under the concentration-time curve over a dosing interval; C_max_, maximum concentration; CSF, cerebrospinal fluid; CV, coefficient of variation; MAD, multiple-ascending dose; NC, not calculable; SAD, single-ascending dose; t_max_, time to maximum observed concentration; t_1/2_, terminal half-life.

^*a*^Mean (CV[%]) values are presented.

^*b*^AUC_0-inf_ for single-dose and AUC_0-tau_ for multiple-dose.

^*c*^Median values are presented.

^*d*^n = 5 due to 1 subject who failed to meet minimum lambda z requirements for the regression.

^*e*^n = 4 due to 2 subjects who failed to meet minimum lambda z requirements for the regression.

^*f*^n = 3 due to 3 subjects who failed to meet minimum lambda z requirements for the regression.

^*g*^n = 2 due to 4 subjects who failed to meet minimum lambda z requirements for the regression.

^*h*^n = 1 due to 5 subjects who failed to meet minimum lambda z requirements for the regression.

**Figure 3.**
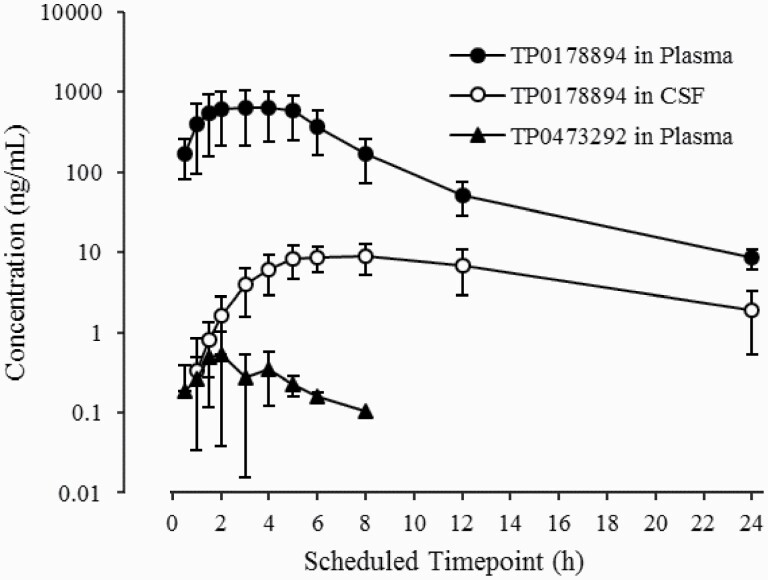
Mean plasma concentration-time profiles of TP0178894 and TP0473292, and cerebrospinal fluid (CSF) concentration-time profiles of TP0178894 following a single oral administration of 100 mg TS-161 under fasted conditions. Figure is presented as the mean ± SD. Figure is presented as the mean +/- SD (n = 6).

Following a single dose of 100 mg TS-161, TP0178894 in CSF reached a mean C_max_ of 9.892 ng/mL at 7 hours post dose, which was delayed by 4 hours compared with that in plasma, and had a mean t_1/2_ of 6 hours (Figure 3; Table 4). TP0178894 penetrated the blood-brain barrier to provide a C_max_ of 1.2% in CSF relative to plasma, slightly higher than the CSF-to-plasma ratio of 0.9% in rats (Table 3).

Urinary excretion of TP0178894 ranged from 20.47% to 79.45% under fasted conditions and 85.48% to 94.01% under fed conditions (supplementary Table 5), while that of TP0473292 was <0.001%. The renal clearance of TP0178894 ranged from 5.262 to 8.868 L/h (supplementary Table 5), which was almost the same as the human glomerular filtration rate (5.88–7.86 L/h) (Koopman et al., 1996). Note that plasma exposures of inactive metabolites, TP0037870 and TP0478768, increased with increasing dose, except plasma TP0037870 exposures in Part C at 150 mg TS-161 (supplementary Figure 1; supplementary Table 6). Urinary excretion of TP0478768 was approximately 50% of the administered doses (supplementary Table 5), while that of TP0037870 was approximately 0.1%, suggesting that TP0037870 was excreted into urine as a glucuronide.

### Safety and Tolerability

Oral administration of TS-161 was safe and well tolerated up to 400 mg in a single dose under fasted conditions and up to 100 mg for 10-day multiple doses under fed conditions in healthy subjects. There were no deaths or serious AEs during the study. Thirty-nine (72.2%) of 54 subjects in the TS-161 group reported 85 AEs, of which 77 (90.6%) events were considered related to study drug. The majority (88.2%) of AEs in the TS-161 group were mild and there were no severe AEs. There were no apparent treatment- or dose-related trends in clinical laboratory tests, vital signs, body weight, electrocardiograms, or physical/neurological examinations nor statistical significance in BPRS, CADSS, or RBANS coding subtest scores, except for 1 timepoint in RBANS coding subtest scores at Part C, Day 1, 6 hours post dose (supplementary Table 7). No changes were reported for Columbia-Suicide Severity Rating Scale. A summary of AEs is shown in Table 5.

**Table 5.** Summary of AEs (2 or More Subjects in Any Dose Level)^*a*^

Part	Part A (SAD)								Part B (CSF)	Part C (MAD)				
Treatment	TS-161							Placebo	TS-161	TS-161				Placebo
Dose	15 mg	50 mg		100 mg	200 mg	400 mg	Combined		100 mg	50 mg	100 mg	150 mg	Combined	
Food condition	Fasted	Fasted	Fed	Fasted					Fasted	Fed				
n	6	6	6	6	6	6	30	10	6	6	6	6	18	6
Total no. of AEs	3	4	7	4	5	9	32	11	8	5	12	28	45	9
No. of participants with at least 1 AE	2 (33.3)	1 (16.7)	3 (50.0)	3 (50.0)	5 (83.3)	5 (83.3)	18 (60.0)	4 (40.0)	6 (100)	4 (66.7)	6 (100)	5 (83.3)	15 (83.3)	3 (50.0)
Somnolence	1 (16.7)	0	1 (16.7)	0	0	1 (16.7)	3 (10.0)	2 (20.0)	0	2 (33.3)	2 (33.3)	0	4 (22.2)	1 (16.7)
Dizziness	0	0	0	0	0	2 (33.3)	2 (6.7)	1 (10.0)	0	1 (16.7)	0	1 (16.7)	2 (11.1)	0
Dizziness postural	0	0	0	0	0	0	0	0	0	0	0	2 (33.3)	2 (11.1)	0
Cognitive disorder	0	0	2 (33.3)	0	0	0	2 (6.7)	0	0	0	0	0	0	0
Headache	0	1 (16.7)	0	0	0	0	1 (3.3)	1 (10.0)	0	0	1 (16.7)	3 (50.0)	4 (22.2)	1 (16.7)
Disturbance in attention	0	0	0	0	0	0	0	0	0	0	0	2 (33.3)	2 (11.1)	1 (16.7)
Orthostatic heart rate response increased	0	1 (16.7)	0	2 (33.3)	0	1 (16.7)	4 (13.3)	0	0	0	0	1 (16.7)	1 (5.6)	2 (33.3)
Orthostatic hypotension	0	0	1 (16.7)	2 (33.3)	0	1 (16.7)	4 (13.3)	1 (10.0)	0	0	0	0	0	0
Nausea	0	0	0	0	0	2 (33.3)	2 (6.7)	0	0	1 (16.7)	2 (33.3)	5 (83.3)	8 (44.4)	0
Vomiting	0	0	0	0	0	0	0	0	0	0	0	3 (50.0)	3 (16.7)	0
Constipation	0	0	0	0	0	0	0	0	0	0	0	2 (33.3)	2 (11.1)	0
Abnormal dreams	0	0	0	0	1 (16.7)	0	1 (3.3)	1 (10.0)	0	0	1 (16.7)	2 (33.3)	3 (16.7)	0
Post lumbar puncture syndrome	0	0	0	0	0	0	0	0	5 (83.3)	0	0	0	0	0

Abbreviations: AE, adverse event; CSF, cerebrospinal fluid; MAD, multiple-ascending dose; SAD, single-ascending dose.

^*a*^Values are shown as number of subjects (%). AE terms are presented per MedDRA Preferred Terms.

In Part A, the total number of AEs and the number of subjects experiencing AEs increased with increasing dose levels; however, there were no other apparent dose-related or food condition-related trends in the incidence of individual AEs. In Part C, 2 subjects on 150 mg TS-161 (Cohort 8) prematurely discontinued related to AEs and withdrew after dosing on Day 1; the AEs were mild or moderate in intensity. The most frequent AEs limiting the tolerability of 150 mg TS-161 under fed conditions were nausea and vomiting. The dose level for Cohort 9 was subsequently reduced to 100 mg TS-161 following completion of Cohort 8 at 150 mg TS-161, which was well tolerated with no vomiting or subject withdrawal.

## Discussion

In the present studies, we demonstrate that (1) TP0178894 is a potent and selective mGlu2/3 receptor antagonist with (*R*,*S*)-ketamine-like antidepressant effects in animal models, (2) TS-161 is orally bioavailable in healthy subjects and is extensively converted into the active metabolite TP0178894, (3) oral TS-161 administration results in a CSF concentration of TP0178894 sufficient to exert pharmacological effects, and (4) TS-161 is safe and generally well tolerated in healthy subjects.

In previous preclinical studies, we reported that mGlu2/3 receptor antagonists exert antidepressant-like effects in rodents similar to those of (*R*,*S*)-ketamine (Chaki, 2017). In this study, we confirmed that oral administration of a prodrug, TP0473292, like other mGlu2/3 receptor antagonists, exhibited antidepressant-like effects in animal models, including one refractory to currently prescribed antidepressants. We also confirmed that TP0473292 is rapidly and completely converted into TP0178894 after oral administration in rats, and oral administration of TP0473292 attenuated mGlu2/3 receptor agonist-induced behavioral changes. Notably, TP0473292 exerted antidepressant-like effects in rodent models at doses that give a CSF concentration of TP0178894 equal to or greater than its K_i_ values of affinities for mGlu2/3 receptors. Collectively, TP0473292 exerts the effects via blockade of mGlu2/3 receptor in vivo by its active metabolite, TP0178894.

Previously, we demonstrated a successful prodrug strategy in which an ester-based lipophilic prodrug of a glutamate analogue (TS-134) achieved good gastrointestinal absorption but also reduced the systemic exposure to the prodrug itself in humans (Watanabe et al., 2020). We took advantage of the same prodrug strategy to synthesize TP0473292 (TS-161) and confirmed that the plasma exposure of TP0473292 in humans was minimal over the dose range tested with or without food intake, in which the C_max_ and AUC of TP0473292 were approximately 0.1% of those of TP0178894 in a molar concentration. In addition, food increased the plasma exposure of TP0178894, which is often seen for lipophilic, poorly water-soluble compounds like TP0473292, and is expected to enhance their oral bioavailability (Wu and Benet, 2005; Koziolek et al., 2019). Indeed, the urinary excretion of TP0178894, which closely reflects the absorbed amount of TP0473292, increased under a fed condition and ranged over 85% of the administered dose, demonstrating good gastrointestinal absorption of TP0473292. Thus, we confirmed that TP0473292 is well absorbed and almost completely converted into TP0178894 by first-pass metabolism in humans. Note that TP0473292 functioned as an ideal prodrug in the viewpoint of PK compared with our previous lead MGS0210 (BCI-838), a prodrug of an mGlu2/3 receptor antagonist MGS0039 (BCI-632), for which plasma exposure was approximately 10-fold higher than that of the active metabolite MGS0039 (Gadient et al., 2012).

The mean CSF C_max_ of TP0178894 after administration of 100 mg TS-161 under fasted conditions in humans was 9.892 ng/mL (30.2 nmol/L), which is comparable with the CSF concentration of TP0178894 (5.74 ng/mL [17.5 nmol/L]) (Table 3) after oral administration of TP0473292 at an effective dose in rodent models. Notably, the mean CSF C_max_ value in humans is also comparable with the IC_50_ value of antagonist activity of TP0178994 at the mGlu2 receptor (23.3 nmol/L) and mGlu3 receptor (20.9 nmol/L) and is approximately 7- to 10-fold higher than the K_i_ values (affinity for mGlu2/3 receptors) of TP0178894. Therefore, CSF exposure of TP0178894 after oral administration of TS-161 provides sufficient inhibition of the mGlu2/3 receptor to exert its pharmacological effects in humans. Since we observed the positive food effect on the plasma exposures of TP0178894, the CSF concentrations of TP0178894 under fed conditions are also expected to increase at a similar extent to plasma. Although the CSF concentrations of TP0178894 were measured only at the 100 mg-dose level, the dose-dependent increase in the CSF exposure of TP0178894 was confirmed in rats at the comparable plasma exposure levels with those in humans (Table 3); therefore, CSF concentrations of TP0178894 in humans are also expected to be dose dependent over the dose range tested.

The most frequently observed AEs with exposure-related incidence during the study overall were nausea, vomiting, and dizziness (including dizziness postural). Nausea was observed in 83% and vomiting in 50% of subjects who received 150 mg TS-161 under fed conditions in Part C (MAD), the TS-161 group that had the highest plasma exposures in the study. The CSF C_max_ of TP0178894 after the repeat dosing of 150 mg TS-161 under fed conditions was estimated to be approximately 95 nmol/L, which is approximately four- to five-fold higher than the IC_50_ value of antagonist activity of TP0178994 at the mGlu2 receptor (23.3 nmol/L) and the mGlu3 receptor (20.9 nmol/L). It is of note that none of these exposure-related AEs were observed at the dose levels of 100 mg TS-161 or lower under fasted conditions in healthy volunteers in which the mean CSF C_max_ value was comparable with the IC_50_ value of antagonist activity of TP0178994 at the mGlu2/3 receptors. The onset time of these AEs varied with no clear relationship with individual PK profiles, and the underlying mechanism of vomiting and nausea that occurred with TS-161 is currently unclear. Selective serotonin reuptake inhibitor–induced vomiting and nausea are probably due to increases in serotonin release in the gastrointestinal tract and the brainstem. Although we previously reported that an mGlu2/3 receptor antagonist increases serotonin transmission in the medial prefrontal cortex (Chaki and Fukumoto, 2019), it is not known if this mechanism is responsible for vomiting and nausea. However, the reduced dose level of 100 mg TS-161 under fed conditions, which is consistent with the expected TP0178894 exposure to exert sufficient pharmacological effects in humans, was well tolerated with only mild AEs, no incidence of vomiting, and no subject withdrawals, supporting continued clinical development of TS-161. The Phase 2 study evaluating the efficacy and safety of TS-161 in patients with TRD is currently on-going with a top dose level of 100 mg/d (ClinicalTrials.gov identifier: NCT04821271) based on the results from the Phase 1 study.

In conclusion, the prodrug TP0473292, orally administered as TS-161, was safe and well tolerated up to 400 mg in single doses under fasted conditions and up to 100 mg for 10-day multiple doses under fed conditions in healthy subjects. A successful prodrug strategy in humans was confirmed, where the prodrug TP0473292 was rapidly converted into the active metabolite TP0178894, which penetrated into the CSF to act on presumed drug targets. These results warrant further clinical development of TS-161 in a patient population with depression, including TRD.

## Supplementary Material

pyab062_suppl_Supplementary_Materials

## Interest Statement

M. Watanabe and B. Marcy are full-time employees of Taisho Pharmaceutical R&D Inc. A. Hiroki, H. Watase, K. Kinoshita, M.Iijima, T. Marumo, and S. Chaki are full-time employees of Taisho Pharmaceutical Co., Ltd. C. Zarate is a full-time employee of the Intramural Research Program at the National Institute of Mental Health, National Institutes of Health (IRP-NIMH-NIH; ZIAMH002927). C. Zarate is listed as a coinventor on a patent for the use of ketamine in major depression and suicidal ideation. C. Zarate is listed as a coinventor on a patent for the use of (2*R*,6*R*)-hydroxynorketamine, (*S*)-dehydronorketamine and other stereoisomeric dehydro and hydroxylated metabolites of (*R*,*S*)-ketamine metabolites in the treatment of depression and neuropathic pain. C. Zarate is listed as co-inventor on a patent application for the use of (2*R*,6*R*)-hydroxynorketamine and (2*S*,6*S*)-hydroxynorketamine in the treatment of depression, anxiety, anhedonia, suicidal ideation, and post-traumatic stress disorders. C. Zarate has assigned his patent rights to the US government but will share a percentage of any royalties that may be received by the government.
