# Supplementary material for: Evaluation of the Safety, Tolerability, and Pharmacokinetic Profiles of TP0473292 (TS-161), A Prodrug of a Novel Orthosteric mGlu2/3 Receptor Antagonist TP0178894, in Healthy Subjects and Its Antidepressant-Like Effects in Rodents
Source: Int J Neuropsychopharmacol. 2021 Sep 17;25(2):106–17. doi: 10.1093/ijnp/pyab062 (PMC8832229; doi:10.1093/ijnp/pyab062)
Supplement: pyab062_suppl_Supplementary_Materials [file pyab062_suppl_supplementary_materials.doc]

**SUPPLEMENTARY MATERIALS**

**Materials and methods for in vitro pharmacology studies**

**Materials:** [^3^H]LY341495 (specific radioacticity: 1.48 TBq/mmol) and [^35^S]guanosine 5’-(γ-thio)triphosphate (GTPγS) (specific radioactivity: 46.2 TBq/mmol) were purchased from American Radiolabeled Chemicals, Inc. (Saint Louise, MO, USA) and PerkinElmer Japan Co., Ltd (Yokohama, Kanagawa, Japan), respectively. Chinese hamster ovary (CHO)-K1 cells stably expressing human mGlu2 receptor and CHO cells stably expressing human mGlu3 receptor were established at a Research Center of Taisho Pharmaceutical Co., Ltd, and CHO cells stably expressing rat mGlu2 or rat mGlu3 receptor were kindly provided from Dr. Shigetada Nakanishi at Kyoto University. For in vitro studies, TP0178894 was dissolved in 0.1 mol/L NaOH and diluted with respective assay buffers, and TP0473292 was dissolved in dimethyl sulfoxide and diluted with respective assay buffers.

**In vitro pharmacological studies:**

Antagonist activities for other mGlu receptors: Antagonist activities of TP0178894 for human mGlu1, human mGlu5 and human mGlu7 receptors were evaluated by glutamate (L-AP4 for mGlu7)-increased [Ca^2+^]i assay, and antagonists activities for human mGlu4, human mGlu6 and human mGlu8 receptors were evaluated by glutamate-increased [^35^S]GTPγS binding assay. Brief conditions for these assays were summarized in Supplementary Table 1.

Selectivity over other receptors, transporters and ion channels: Both TP0178894 and TP0473292 were run against a panel of 66 receptor binding assays (Eurofin Cerep, Le Bois l’Evêque, France) according to previously reported methods. Both TP0178894 and TP0473292 were tested in duplicate at 10,000 nmol/L.

**Statistical analysis for clinical study**

**Statistical analysis for BPRS, CADSS, and Coding subtest of RBANS:** Treatment effect was analyzed using analysis of covariance with treatment group as fixed effect and baseline as covariate at specific post baseline visit and timepoint. Subjects treated with TS-161 were pooled together for each treatment comparison, as were placebo subjects.

**Supplementary Table 1. Assay conditions and antagonist activities of TP0178894 for mGlu1, mGlu4, mGlu5, mGlu6, mGlu7 and mGlu8 receptor subtypes**

Data represent mean [95% confidence interval] obtained from 3 to 4 independent experiments.

**Supplementary Table 2. Selectivity of TP0178894 and TP0473292 over 66 receptors, transporters and ion channels**

Effects of TP0178894 (10,000 nmol/L) or TP0473292 (10,000 nmol/L) for 66 receptors, transporters and ion channels were evaluated by respective receptor binding assays. Values represent mean % inhibition of each receptor binding, done in duplicate.

**Supplementary Table 3. Subject Disposition**

| **Part** | **Part A (SAD)** | | | | | | | | | | **Part B (CSF)** | **Part C (MAD)** | | | | | |
| --- | --- | --- | --- | --- | --- | --- | --- | --- | --- | --- | --- | --- | --- | --- | --- | --- | --- |
| **Treatment** | **TS-161** | | | | | | | **Placebo^a^** | | **Total** | **TS-161** | **TS-161** | | | | **Placebo** | **Total** |
| **Dose** | **15 mg** | **50 mg^a^** | | **100 mg** | **200 mg** | **400 mg** | **Combined** |  |  |  | **100 mg** | **50 mg** | **100 mg** | **150 mg** | **Combined** |  |  |
| **Food Condition** | **Fasted** | **Fasted** | **Fed** | **Fasted** | | |  | **Fasted** | **Fed** |  | **Fasted** | **Fed** | | | | | |
| **Randomized** | 6 | 6 | 6 | 6 | 6 | 6 | 30 | 10 | 2 | 40 | 6 | 6 | 6 | 6 | 18 | 6 | 24 |
| **Completed** | 6 | 6 | 6 | 6 | 6 | 6 | 30 | 10 | 2 | 40 | 6 | 6 | 6 | 4 | 16 | 5 | 21 |
| **Withdrawal** | 0 | 0 | 0 | 0 | 0 | 0 | 0 | 0 | 0 | 0 | 0 | 0 | 0 | 2 | 2 | 1 | 3 |
| **Safety Population** | 6 | 6 | 6 | 6 | 6 | 6 | 30 | 10 | 2 | 40 | 6 | 6 | 6 | 6 | 18 | 6 | 24 |
| **PK Population** | 6 | 6 | 6 | 6 | 6 | 6 | 30 | 0 | 0 | 30 | 6 | 6 | 6 | 6 | 18 | 0 | 18 |

Values are shown as number of subjects.

CSF, cerebrospinal fluid; MAD, multiple-ascending dose; PK, pharmacokinetic; SAD, single-ascending dose.

^a^ In Part A (SAD) Cohort 2, where 6 subjects were dosed with 50 mg TS-161 and 2 subjects with placebo, the subjects were first dosed under fasted conditions, followed by a washout period, and the second dose under fed conditions.

**Supplementary Table 4.** Subject Demographics

|  | | **Part A (SAD)** | | | | | | | | **Part B (CSF)** | **Part C (MAD)** | | | | | |
| --- | --- | --- | --- | --- | --- | --- | --- | --- | --- | --- | --- | --- | --- | --- | --- | --- |
| **Treatment** | | **TS-161** | | | | | | **Placebo** | **Total** | **TS-161** | **TS-161** | | | | **Placebo** | **Total** |
| **Dose** | | **15 mg** | **50 mg** | **100 mg** | **200 mg** | **400 mg** | **Combined** |  |  | **100 mg** | **50 mg** | **100 mg** | **150 mg** | **Combined** |  |  |
| **Age (years) ^#^** | | 36.7  (7.0) | 28.7  (7.0) | 34.8  (12.1) | 37.8  (7.7) | 37.3  (14.5) | 35.1  (10.0) | 41.1  (8.3) | 36.6  (9.9) | 33.2  (7.6) | 34.7  (5.8) | 39.2  (6.7) | 38.5  (10.0) | 37.4  (7.5) | 31.7  (5.5) | 36.0  (7.4) |
| **Sex n (%)** | **Female** | 2  (33.3) | 2  (33.3) | 1  (16.7) | 5  (83.3) | 4  (66.7) | 14  (46.7) | 7  (70.0) | 21  (52.5) | 3  (50.0) | 2  (33.3) | 4  (66.7) | 4  (66.7) | 10  (55.6) | 4  (66.7) | 14  (58.3) |
|  | **Male** | 4  (66.7) | 4  (66.7) | 5  (83.3) | 1  (16.7) | 2  (33.3) | 16  (53.3) | 3  (30.0) | 19  (47.5) | 3  (50.0) | 4  (66.7) | 2  (33.3) | 2  (33.3) | 8  (44.4) | 2  (33.3) | 10  (41.7) |
| **Ethnicity n (%)** | **Hispanic or Latino** | 1  (16.7) | 0 | 1  (16.7) | 1  (16.7) | 3  (50.0) | 6  (20.0) | 1  (10.0) | 7  (17.5) | 1  (16.7) | 1  (16.7) | 3  (50.0) | 2  (33.3) | 6  (33.3) | 1  (16.7) | 7  (29.2) |
|  | **Not Hispanic or Latino** | 5  (83.3) | 6  (100) | 5  (83.3) | 5  (83.3) | 3  (50.0) | 24  (80.0) | 9  (90.0) | 33  (82.5) | 5  (83.3) | 5  (83.3) | 3  (50.0) | 4  (66.7) | 12  (66.7) | 5  (83.3) | 17  (70.8) |
| **Race n (%)** | **White** | 2  (33.3) | 2  (33.3) | 3  (50.0) | 3  (50.0) | 5  (83.3) | 15  (50.0) | 5  (50.0) | 20  (50.0) | 2  (33.3) | 3  (50.0) | 1  (16.7) | 4  (66.7) | 8  (44.4) | 4  (66.7) | 12  (50.0) |
|  | **Asian** | 1  (16.7) | 0 | 1  (16.7) | 0 | 0 | 2  (6.7) | 1  (10.0) | 3  (7.5) | 2  (33.3) | 0 | 0 | 0 | 0 | 0 | 0 |
|  | **Black or African American** | 3  (50.0) | 4  (66.7) | 1  (16.7) | 3  (50.0) | 1  (16.7) | 12  (40.0) | 1  (10.0) | 13  (32.5) | 2  (33.3) | 3  (50.0) | 3  (50.0) | 2  (33.3) | 8  (44.4) | 2  (33.3) | 10  (41.7) |
|  | **Other** | 0 | 0 | 1  (16.7) | 0 | 0 | 1  (3.3) | 3  (30.0) | 4  (10.0) | 0 | 0 | 2  (33.3) | 0 | 2  (11.1) | 0 | 2  (8.3) |
| **Height (cm) ^#^** | | 175.7  (13.2) | 172.5  (9.3) | 172.2  (5.6) | 165.7  (8.6) | 167.2  (5.0) | 170.6  (9.0) | 167.7  (12.2) | 169.9  (9.8) | 168.3  (12.3) | 175.7  (12.6) | 170.2  (6.5) | 166.2  (10.1) | 170.7  (10.2) | 172.5  (8.1) | 171.1  (9.6) |
| **Weight (kg) ^#^** | | 80.2  (14.6) | 71.5  (10.4) | 77.1  (6.1) | 68.6  (7.7) | 72.0  (13.0) | 73.9  (10.9) | 72.8  (17.5) | 73.6  (12.6) | 72.8  (11.9) | 80.8  (6.9) | 78.9  (9.9) | 67.6  (12.2) | 75.8  (11.1) | 72.9  (11.1) | 75.0  (10.9) |
| **BMI (kg/m^2^) ^#^** | | 25.9  (2.6) | 24.2  (4.2) | 26.0  (1.6) | 25.0  (1.9) | 25.7  (4.2) | 25.3  (3.0) | 25.5  (2.9) | 25.4  (2.9) | 25.7  (3.2) | 26.3  (1.8) | 27.2  (2.4) | 24.4  (2.5) | 25.9  (2.4) | 24.4  (3.2) | 25.6  (2.7) |

^#^ Mean (SD).

BMI, body mass index; SAD, single-ascending dose; MAD, multiple-ascending dose; CSF, cerebrospinal fluid.

**Supplementary Table 5. Urine pharmacokinetic parameters of TP0178894 and TP0478768 by treatment**

1. TP0178894

| Parameter  (unit) | Part A (SAD) | | | | | | Part C (MAD) (Day 10) | | |
| --- | --- | --- | --- | --- | --- | --- | --- | --- | --- |
|  | 15 mg  Fasted  (n=6) | 50 mg  Fasted  (n=6) | 50 mg  Fed  (n=6) | 100 mg  Fasted  (n=6) | 200 mg  Fasted  (n=6) | 400 mg  Fasted  (n=6) | 50 mg  Fed  (n=6) | 100 mg  Fed  (n=6) | 150 mg  Fed  (n=6) |
| Fe (%)^a^ | 79.45  (29.4) | 52.60  (38.7) | 94.01  (24.3) | 34.59  (40.2) | 31.77  (33.8) | 20.47  (30.1) | 88.63  (16.4) | 89.60  (27.5) | 85.48^c^  (14.7) |
| CLr (L/h) | 7.012  (16.6) | 7.689  (24.9) | 8.868^b^  (11.7) | 5.956  (17.2) | 5.262  (33.3) | 5.604  (28.9) | 6.357  (25.4) | 6.505  (32.2) | 7.758^c^  (14.0) |

1. TP0478768

| Parameter  (unit) | Part A (SAD) | | | | | | Part C (MAD) (Day 10) | | |
| --- | --- | --- | --- | --- | --- | --- | --- | --- | --- |
|  | 15 mg  Fasted  (n=6) | 50 mg  Fasted  (n=6) | 50 mg  Fed  (n=6) | 100 mg  Fasted  (n=6) | 200 mg  Fasted  (n=6) | 400 mg  Fasted  (n=6) | 50 mg  Fed  (n=6) | 100 mg  Fed  (n=6) | 150 mg  Fed  (n=6) |
| Fe (%)^a^ | 55.62  (14.8) | 43.74  (26.9) | 48.56  (26.4) | 39.97  (22.4) | 40.55  (18.8) | 30.51  (24.0) | 49.40  (20.3) | 43.88  (32.9) | 57.14^c^  (14.6) |
| CLr (L/h) | 21.120  (18.5) | 21.320  (27.7) | 25.030^b^  (9.4) | 18.960^b^  (7.0) | 15.490  (29.2) | 20.050^b^  (13.7) | 22.250  (34.8) | 15.890  (29.9) | 24.370^c^  (7.8) |

Mean (CV[%]) values are presented.

^a^ Fe_(0-48)_ for Part A and Fe_(0-24)_ for Part C.

^b^ n=5 due to one subject who failed to meet minimum lambda z requirements for the regression.

^c^ n=4 due to two subjects who failed to meet minimum lambda z requirements for the regression.

CLr, renal clearance; CV, coefficient of variation; Fe_(0-x)_, percentage of the amount of drug excreted in urine between time 0 and X; MAD, multiple-ascending dose; SAD, single-ascending dose.

**Supplementary Table 6. Plasma pharmacokinetic parameters of TP0037870 and TP0478768 by treatment**

1. TP0037870 (plasma)

| Parameter  (unit) | Part A (SAD) | | | | | | Part C (MAD) (Day 10) | | |
| --- | --- | --- | --- | --- | --- | --- | --- | --- | --- |
|  | 15 mg  Fasted  (n=6) | 50 mg  Fasted  (n=6) | 50 mg  Fed  (n=6) | 100 mg  Fasted  (n=6) | 200 mg  Fasted  (n=6) | 400 mg  Fasted  (n=6) | 50 mg  Fed  (n=6) | 100 mg  Fed  (n=6) | 150 mg  Fed  (n=6) |
| C_max_  (ng/mL)^a^ | 14.66  (39.9) | 36.30  (51.3) | 41.48  (44.1) | 45.78  (67.4) | 74.62  (32.2) | 104.6  (23.5) | 28.50  (45.5) | 105.4  (42.3) | 102.0^e^  (21.2) |
| AUC  (h•ng/mL)^a,b^ | 43.70^f^  (38.3) | 308.6^h^  (NC) | 178.5^f^  (58.7) | 502.1^g^  (30.7) | 1101^d^  (39.8) | 1188^h^  (NC) | 230.3^d^  (44.8) | 484.5  (37.7) | 476.1^e^  (39.4) |
| t_max_ (h)^c^ | 4.00 | 3.56 | 5.00 | 2.50 | 3.51 | 2.02 | 4.99 | 3.50 | 4.03^e^ |
| t_1/2_ (h)^a^ | 1.401^f^  (51.8) | 10.45^h^  (NC) | 2.088^f^  (35.5) | 7.620^g^  (52.7) | 10.83^d^  (2.2) | 11.39^h^  (NC) | 3.521^f^  (8.4) | 4.671^f^  (22.7) | 6.229^f^  (4.3) |

1. TP0478768 (plasma)

| Parameter  (unit) | Part A (SAD) | | | | | | Part C (MAD) (Day 10) | | |
| --- | --- | --- | --- | --- | --- | --- | --- | --- | --- |
|  | 15 mg  Fasted  (n=6) | 50 mg  Fasted  (n=6) | 50 mg  Fed  (n=6) | 100 mg  Fasted  (n=6) | 200 mg  Fasted  (n=6) | 400 mg  Fasted  (n=6) | 50 mg  Fed  (n=6) | 100 mg  Fed  (n=6) | 150 mg  Fed  (n=6) |
| C_max_  (ng/mL)^a^ | 82.03  (33.5) | 130.2  (35.3) | 144.3  (39.4) | 222.6  (76.6) | 384.0  (17.3) | 529.5  (31.2) | 116.7  (24.6) | 428.0  (22.9) | 619.5^e^  (14.3) |
| AUC  (h•ng/mL)^a,b^ | 286.1^e^  (24.0) | 712.0  (21.3) | 738.0^e^  (27.3) | 1588^f^  (20.2) | 3817  (18.9) | 4794^f^  (36.8) | 775.5  (20.1) | 1857  (21.0) | 2394^e^  (22.8) |
| t_max_ (h)^c^ | 4.00 | 3.56 | 5.00 | 2.26 | 3.50 | 2.02 | 3.55 | 3.50 | 4.50^e^ |
| t_1/2_ (h)^a^ | 4.741^e^  (94.5) | 7.024  (31.6) | 3.036^e^  (18.9) | 6.637^f^  (41.9) | 9.510  (15.6) | 7.812^f^  (67.0) | 4.179  (39.0) | 7.033^d^  (59.3) | 5.802^f^  (14.2) |

^a^ mean (CV[%]) values are presented.

^b^ AUC_0-inf_ for single-dose and AUC_0-tau_ for multiple-dose.

^c^ median values are presented.

^d^ n=5 due to one subject who failed to meet minimum lambda z requirements for the regression.

^e^ n=4 due to two subjects who failed to meet minimum lambda z requirements for the regression.

^f^ n=3 due to three subjects who failed to meet minimum lambda z requirements for the regression.

^g^ n=2 due to four subjects who failed to meet minimum lambda z requirements for the regression.

^h^ n=1 due to five subjects who failed to meet minimum lambda z requirements for the regression.

AUC_0-inf_, area under the concentration-time curve extrapolated to infinity; AUC_0-tau_, area under the concentration-time curve over a dosing interval; C_max_, maximum concentration; CV, coefficient of variation; h, hours; MAD, multiple-ascending dose; NC, not calculable; SAD, single-ascending dose; t_max_, time to maximum observed concentration; t_1/2_, terminal half-life.

**Supplementary Table 7.** Assessment of treatment effects for BPRS, CADSS, and RBANS coding subtest scores

| Assessment | Day | Timepoint (postdose) | TS-161 | | | Placebo | | | TS-161 - Placebo | | |
| --- | --- | --- | --- | --- | --- | --- | --- | --- | --- | --- | --- |
|  |  |  | N | LSM | 90% CI | N | LSM | 90% CI | Mean Difference | 90% CI | P-value |
| BPRS | Day 1 | 3h | 30 | 24.2 | 23.87, 24.48 | 10 | 24.8 | 24.27, 25.32 | -0.6 | -1.23, -0.01 | 0.0932 |
| (Part A) | Day 1 | 6h | 30 | 24.3 | 23.93, 24.64 | 10 | 24.3 | 23.65, 24.89 | 0.0 | -0.70, 0.73 | 0.9684 |
|  | Day 1 | 24h | 30 | 24.3 | 24.06, 24.47 | 10 | 24.3 | 23.91, 24.62 | 0.0 | -0.41, 0.41 | 0.9893 |
|  | Day 3 |  | 30 | 24.1 | 23.85, 24.28 | 10 | 24.4 | 24.02, 24.77 | -0.3 | -0.76, 0.10 | 0.2040 |
| BPRS | Day 1 | 3h | 18 | 24.3 | 24.00, 24.57 | 6 | 24.5 | 23.99, 24.99 | -0.2 | -0.79, 0.37 | 0.5452 |
| (Part C) | Day 1 | 6h | 18 | 24.1 | 23.99, 24.21 | 6 | 24.0 | 23.85, 24.24 | 0.1 | -0.17, 0.28 | 0.6787 |
|  | Day 4 |  | 16 | 25.1 | 24.27, 25.98 | 5 | 24.8 | 23.26, 26.35 | 0.3 | -1.46, 2.10 | 0.7594 |
|  | Day 10 | 3h | 16 | 24.3 | 23.99, 24.61 | 5 | 24.0 | 23.48, 24.61 | 0.3 | -0.40, 0.90 | 0.5094 |
|  | Day 10 | 6h | 16 | 24.3 | 24.07, 24.55 | 5 | 24.0 | 23.58, 24.45 | 0.3 | -0.21, 0.79 | 0.3205 |
|  | Day 10 | 24h | 16 | 24.3 | 24.04, 24.59 | 5 | 24.4 | 23.88, 24.88 | -0.1 | -0.63, 0.52 | 0.8611 |
|  | Day 10 | 48h | 16 | 24.2 | 24.09, 24.28 | 5 | 24.2 | 24.04, 24.39 | 0.0 | -0.23, 0.17 | 0.7747 |
| CADSS | Day 1 | 3h | 30 | 0.0 | NC | 10 | 0.0 | NC | 0.0 | NC | 1.0000 |
| (Part A) | Day 1 | 6h | 30 | 0.1 | -0.05, 0.25 | 10 | 0.0 | -0.26, 0.26 | 0.1 | -0.19, 0.39 | 0.5705 |
|  | Day 1 | 24h | 30 | 0.0 | NC | 10 | 0.0 | NC | 0.0 | NC | 1.0000 |
|  | Day 3 |  | 30 | 0.0 | NC | 10 | 0.0 | NC | 0.0 | NC | 1.0000 |
| CADSS | Day 1 | 3h | 18 | 0.0 | NC | 6 | 0.0 | NC | 0.0 | NC | 1.0000 |
| (Part C) | Day 1 | 6h | 18 | 0.0 | NC | 6 | 0.0 | NC | 0.0 | NC | 1.0000 |
|  | Day 4 |  | 16 | 0.5 | -0.10, 1.10 | 5 | 0.0 | -1.06, 1.06 | 0.5 | -0.72, 1.72 | 0.4869 |
|  | Day 10 | 3h | 16 | 0.0 | NC | 5 | 0.0 | NC | 0.0 | NC | 1.0000 |
|  | Day 10 | 6h | 16 | 0.1 | -0.03, 0.16 | 5 | 0.0 | -0.17, 0.17 | 0.1 | -0.13, 0.26 | 0.5893 |
|  | Day 10 | 24h | 16 | 0.0 | NC | 5 | 0.0 | NC | 0.0 | NC | 1.0000 |
|  | Day 10 | 48h | 16 | 0.0 | NC | 5 | 0.0 | NC | 0.0 | NC | 1.0000 |
| RBANS | Day 1 | 3h | 30 | 51.1 | 49.42, 52.77 | 10 | 47.9 | 45.01, 50.82 | 3.2 | -0.17, 6.53 | 0.1179 |
| (Part A) | Day 1 | 6h | 30 | 51.2 | 49.65, 52.80 | 10 | 50.3 | 47.61, 53.07 | 0.9 | -2.27, 4.04 | 0.6393 |
|  | Day 1 | 24h | 30 | 54.4 | 52.86, 55.97 | 10 | 53.7 | 50.96, 56.36 | 0.8 | -2.37, 3.87 | 0.6871 |
|  | Day 3 |  | 30 | 55.2 | 53.81, 56.56 | 10 | 52.6 | 50.25, 55.02 | 2.6 | -0.20, 5.31 | 0.1260 |
| RBANS | Day 1 | 3h | 18 | 49.5 | 47.50, 51.53 | 6 | 51.8 | 48.29, 55.30 | -2.3 | -6.33, 1.78 | 0.3449 |
| (Part C) | Day 1 | 6h | 18 | 49.8 | 48.47, 51.20 | 6 | 53.3 | 50.97, 55.70 | -3.5 | -6.24, -0.76 | 0.0391 |
|  | Day 4 |  | 16 | 53.3 | 50.88, 55.72 | 5 | 55.6 | 51.26, 60.02 | -2.3 | -7.38, 2.71 | 0.4317 |
|  | Day 10 | 3h | 16 | 53.1 | 51.24, 54.90 | 5 | 53.6 | 50.26, 56.89 | -0.5 | -4.33, 3.31 | 0.8195 |
|  | Day 10 | 6h | 16 | 53.1 | 52.51, 57.78 | 5 | 54.5 | 49.76, 59.29 | 0.6 | -4.86, 6.11 | 0.8460 |
|  | Day 10 | 24h | 16 | 55.6 | 53.43, 57.75 | 5 | 56.7 | 52.80, 60.62 | -1.1 | -5.62, 3.39 | 0.6719 |
|  | Day 10 | 48h | 16 | 59.1 | 57.13, 61.02 | 5 | 57.8 | 54.25, 61.27 | 1.3 | -2.73, 5.36 | 0.5790 |

N: Number of subjects exposed to each treatment who were included in the mixed model.

BPRS, Brief Psychiatric Rating Scale; CADSS, Clinician Administered Dissociative States Scale; RBANS, The Repeatable Battery for the Assessment of Neuropsychological Status; LSM, least squares mean; CI, confidence interval; NC, not calculated.

**Supplementary Figure 1. Mean plasma concentration-time profiles of inactive metabolites, TP0037870 and TP0478768, following single-ascending dose (SAD) under fasted conditions and multiple-ascending dose (MAD) under fed conditions of TS-161**


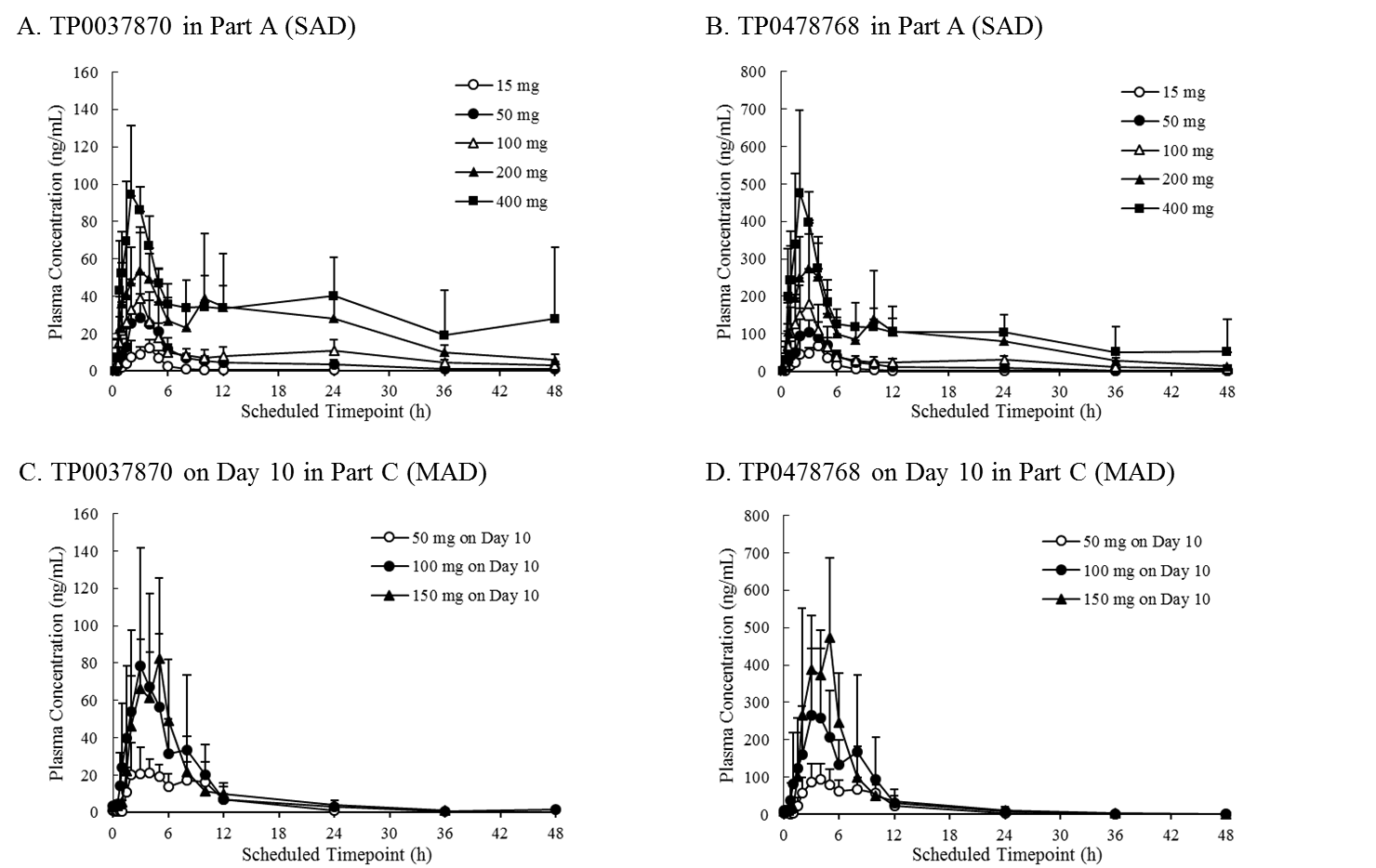


Figures are presented as the mean + standard deviation.
